# The Orphan Adhesion-GPCR GPR126 Is Required for Embryonic Development in the Mouse

**DOI:** 10.1371/journal.pone.0014047

**Published:** 2010-11-18

**Authors:** Helen Waller-Evans, Simone Prömel, Tobias Langenhan, John Dixon, Dirk Zahn, William H. Colledge, Joanne Doran, Mark B. L. Carlton, Ben Davies, Samuel A. J. R. Aparicio, Johannes Grosse, Andreas P. Russ

**Affiliations:** 1 Department of Biochemistry and Magdalen College, University of Oxford, Oxford, United Kingdom; 2 Takeda Cambridge Ltd, Cambridge, United Kingdom; 3 Department of Physiology, Development and Neuroscience, University of Cambridge, Cambridge, United Kingdom; 4 Wellcome Trust Centre for Human Genetics, University of Oxford, Oxford, United Kingdom; 5 Department of Pathology and Laboratory Medicine and BC Cancer Research Centre, University of British Columbia, Vancouver, Canada; University College London, United Kingdom

## Abstract

Adhesion-GPCRs provide essential cell-cell and cell-matrix interactions in development, and have been implicated in inherited human diseases like Usher Syndrome and bilateral frontoparietal polymicrogyria. They are the second largest subfamily of seven-transmembrane spanning proteins in vertebrates, but the function of most of these receptors is still not understood. The orphan Adhesion-GPCR GPR126 has recently been shown to play an essential role in the myelination of peripheral nerves in zebrafish. In parallel, whole-genome association studies have implicated variation at the GPR126 locus as a determinant of body height in the human population. The physiological function of GPR126 in mammals is still unknown. We describe a targeted mutation of GPR126 in the mouse, and show that GPR126 is required for embryonic viability and cardiovascular development.

## Introduction

Adhesion-GPCRs are the second largest subfamily of putatively G-protein coupled receptors (GPCR) with more than 30 members in mammals [1]. Their typical domain architecture consists of a C-terminal seven-transmembrane spanning (7TM) domain homologous to secretin-like GPCRs, and a long N-terminal domain containing a range of protein domains found in cell adhesion proteins [2]. N- and C-terminal domains can be autocatalytically cleaved at the membrane-proximal GPS (GPCR proteolytic site) domain, which is a characteristic feature of this receptor class [3].

The biological function of most Adhesion-GPCRs is still unknown. Mutations in some members of the protein family have been identified as the cause of inherited developmental defects in humans like Usher Syndrome (VLGR1) [4] and bilateral frontoparietal polymicrogyria (GPR56) [5]. The cadherin-like Adhesion-GPCR flamingo (alternative names: stan (starry night); CELSR (cadherin, EGF-like, LAG-like, and seven-pass receptor)) has been implicated in planar cell polarity in Drosophila [6], [7] and axonal tract development in mice [8]. We have recently shown that a highly conserved Adhesion-GPCR, the Latrophilin homolog *lat-1*, is essential for tissue polarity in the *C. elegans* embryo [9]. Although there is no consensus yet about the physiological function of Adhesion-GPCRs and their molecular mechanism of signalling, the existing data suggest that this receptor class mediates essential cell-cell and cell-matrix interactions [2].

Recently, an orphan receptor of the Adhesion-GPCR family, GPR126, has been shown to play an essential role in the myelination of peripheral nerves by neural crest (NC) -derived Schwann cells in the zebrafish *Danio rerio* [10]. Schwann cells lacking GPR126 expression fail to to a myelinate their target axons, suggesting that GPR126 is a key component of the elusive signalling pathway by which axons communicate with the myelinating cell [11], [12].

In mammals, GPR126 has been described as an orphan receptor with a tightly regulated expression pattern in mouse development (DREG) [13], and has also been isolated from human umbilical vein endothelial cell (HUVEC) cultures (vascular inducible GPCR, VIGR) [14]. Surprisingly, whole-genome association studies have identified genetic variation at the GPR126 locus as a determinant of trunk length and body height in the human population [15], [16], [17]. The physiological function of GPR126 in mammals is unknown, and there is currently no model that could explain the molecular mechanism of this trait.

We describe here a targeted mutation of GPR126 in the mouse and show that the receptor is required for embryonic viability and cardiovascular development.

## Results

### GPR126 is an evolutionary innovation specific for vertebrates

**Figure 1:**
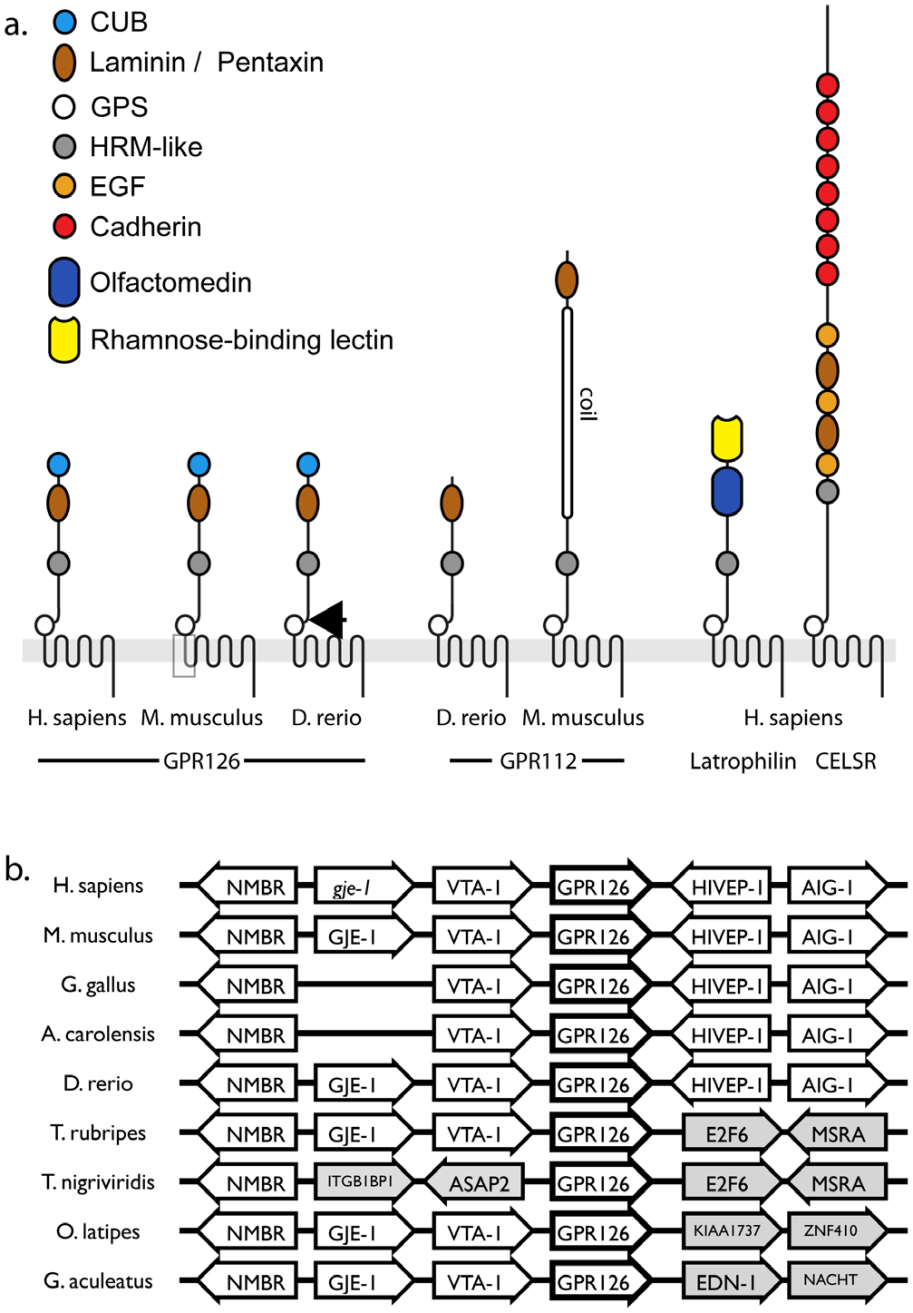
The domain architecture and genomic conservation of GPR126. **a**. The domain architecture of GPR126 in comparison to other Adhesion-GPCRs. Sketches are drawn to scale of primary protein structure, the conserved domains of the N-terminus are colour-coded. The shaded box in mouse GPR126 depicts the region deleted in the targeted allele GPR126^LacZ^. The arrow in zebrafish GPR126 shows the point of truncation in *gpr126(st49)* [10]. The closest homolog of GPR126 is GPR112, which shares the Pentaxin domain but lacks the CUB domain. In zebrafish, GPR126 and GPR112 are structurally more similar than in mammals, where GPR112 contains a very long coiled domain in its N-terminus. CUB: C1r/C1s, Uegf, Bmp1 domain; EGF: Epidermal Growth Factor domain; HRM: Hormone-binding motif. **b**. Schematic depiction of the conserved synteny surrounding the GPR126 locus in diverse vertebrate species. Note that the synteny of the zebrafish locus is highly conserved to reptiles, birds, and mammals, while other fish species show higher divergence. NMBR: neuromedin B receptor; GJE-1: gap junction protein epsilon 1 (pseudogene *gje-1* in humans); VTA-1 Vps20-associated 1 homolog (S. cerevisiae); HIVEP-2: HIV enhancer binding protein 2; AIG-1: androgen-induced gene 1; E2F6: E2F transcription factor 6; MSRA: methionine sulfoxide reductase A; ASAP2; ArfGAP with SH3 domain, ankyrin repeat and PH domain 2; ITGB1BP1: integrin beta 1 binding protein 1: EDN-1; endothelin 1; NACHT: NACHT-NTPase containing protein; KIAA1737: unknown novel protein; ZNF410: zinc-finger protein 410.

The GPR126 locus is highly conserved in vertebrates. We could identify putative genes encoding homologous proteins with the typical domain architecture (CUB, Laminin G/Pentaxin, GPS, 7TM2, Fig. 1a) in all vertebrate species that were investigated. The synteny of the genomic region surrounding these genes is conserved in fish, birds, reptiles, and mammals, indicating that the predicted genes are true orthologues (Fig. 1b). A GPR126 homologue is also present in the amphibian *Xenopus tropicalis*, but although synteny also appears to be conserved a comprehensive annotation is currently not possible due to the fragmentation of sequence contigs.

The genomes of the sea urchin *S. purpuratus* and of *Trichoplax adherens* are predicted to encode Adhesion-GPCRs with N-termini containing a CUB domain in conjunction with EGF or Ig-like domains, respectively. Thus, the domain architectures of these putative receptors are clearly divergent from vertebrate GPR126. No orthologues of GPR126 were found in the high quality genomes of the chordates *Ciona intestinalis*, *C. savignyi* and *Branchiostoma floridae*, or in the draft genome assembly of the lamprey *Petromyzon marinus*.

These results indicate that GPR126 is specific for vertebrates. This would be consistent with the proposed function in Schwann cell precursors (SCP) [10] which originate from the NC. The NC is considered to be a developmental innovation that is unique to vertebrates [18].

The closest paralog of GPR126 is GPR112, which also appears to be specific for vertebrates (Fig. 1a). In mammals, GPR112 is located on the X chromosome and contains a long (>1500 amino acids) domain in its N-terminus predicted to be an extended coil structure. This domain is not present in GPR126. In zebrafish, GPR112 is located on Chromosome 10, is lacking the coil domain, and is highly similar to zebrafish GPR126 (Chr. 20, amino acid identity 30%, similarity 48%, Figure S1).

### A targeted mutation of GPR126 expressing a LacZ reporter gene

**Figure 2:**
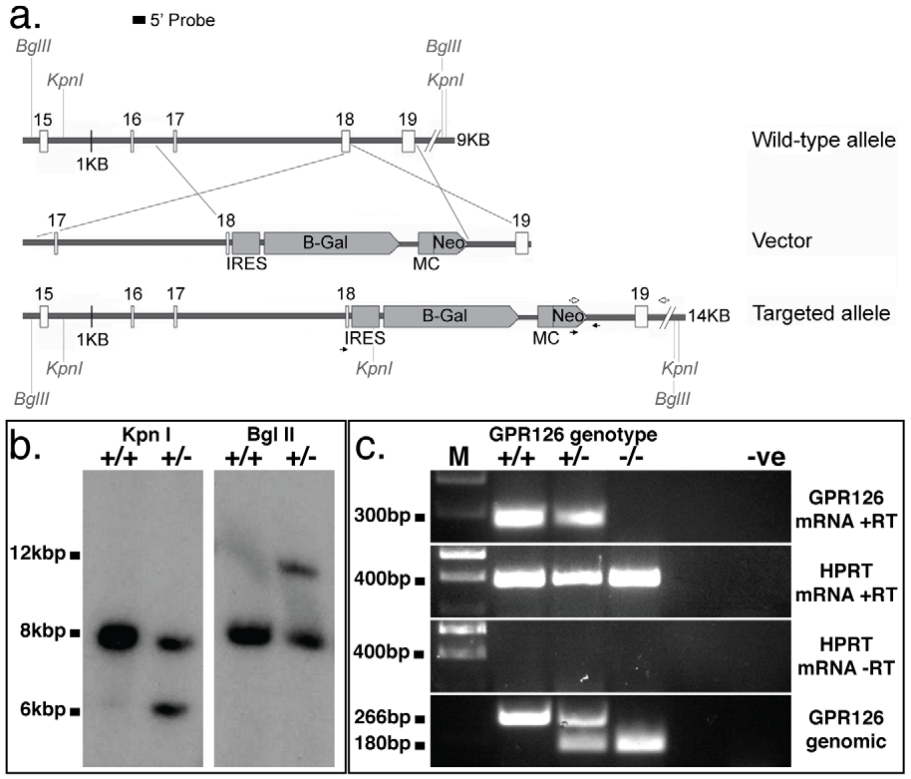
Disruption of the mouse GPR126 gene by homologous recombination in embryonic stem cells. **a**. Schematic depiction of the wild-type GPR126 locus, the targeting vector, and the targeted locus after homologous recombination. Numbered boxes represent GPR126 coding exons 15–19, LacZ reporter and neomycin-resistance gene expression cassettes are shown as shaded boxes. KpnI, and BglII restriction enzymes sites as used in b. Arrows depict PCR primers used in c. b. Southern blot showing correct targeting of the GPR126 locus in embryonic stem cells, using the hybridization probe shown in a. Correct integration creates a new KpnI fragment of ∼6 kb and increases the size of the BglII fragment to ∼ 12 kb. c. PCR analysis demonstrating the absence of GPR126 transcript (top row) and the deletion of the GPR126 genomic locus (bottom row). HPRT transcript was used as a control for cDNA concentration in RT-PCR. The genomic PCR used three primers to amplify the wild-type and mutant fragments in a single reaction. +/+; wild-type samples; +/−: heterozygote samples; −/−: homozygous mutant samples.

To follow the expression pattern of GPR126 in detail and to identify its physiological function we generated a targeted mutation in murine embryonic stem cells which disrupts the GPR126 coding region and expresses a *LacZ* reporter gene under the control of the GPR126 promotor (GPR126^LacZ^). The sequences encoding part of the 7TM domain were deleted (Fig. 1a), and a *LacZ* reporter gene cassette [19] was inserted into the locus (Fig. 2a, b).

Heterozygous GPR126^LacZ^/+ carriers were born at the expected Mendelian frequency (Table 1) and did not show any obvious phenotypes, indicating that the targeted locus does not have detrimental dominant activity. Wild-type GPR126 transcript could not be detected in homozygous embryos (see below), suggesting that the targeted allele is a null mutation (Fig. 2c).

**Table 1:** Live offspring from GPR126^LacZ^ matings.

Stage	Cross	+/+		+/−		−/−		N
	genotypes	n	%	n	%	n	%	
**weanlings**	+/− x wt	186	49.3%	191	50.7%	n/a		377
**weanlings**	+/− x +/−	10	32.3%	21	67.7%	0	0.0%	31

Intercrosses of animals heterozygous for GPR126^LacZ^ do not produce liveborn homozygous offspring.

### Dynamic segmental expression of GPR126 in the embryo

We investigated the expression pattern of the wild-type GPR126 gene in adult tissues by RT-PCR, *in situ* hybridization (ISH), and immunofluorescence staining (IF). Our PCR analysis confirmed that GPR126 mRNA is widely expressed (Figure S2) [13]. However, in spite of substantial efforts we were not able to reproducibly detect the wild-type gene product on a cellular level by ISH or IF.

**Figure 3:**
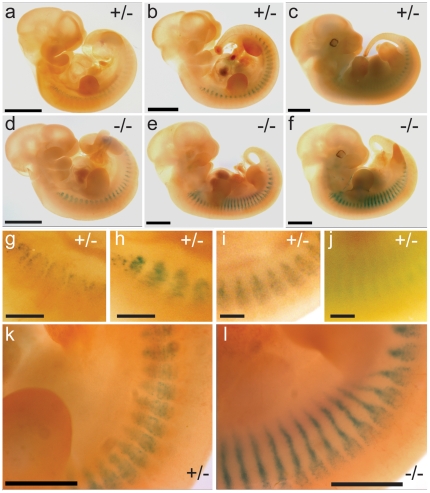
The expression pattern of the GPR126^LacZ^ reporter allele. Expression pattern of GPR126. Embryos carrying the GPR126^LacZ^ allele were stained for β-galactosidase activity as whole-mount preparations. Heterozygous (a–c) and homozygous (d–f) embryos at 10.5, 11.5, and 12.5 dpf, respectively. (g–j): higher magnification view of expression in somitic region in a heterozygous embryo. Note the faint and transient expression. (k, l): comparison of heterozygous and homozygous embryo at 11.5 dpf. Note the change in expression pattern and intensity in the homozygous specimen. Scalebar (a–f) 2 mm, (g–l) 500 µm.

**Figure 4:**
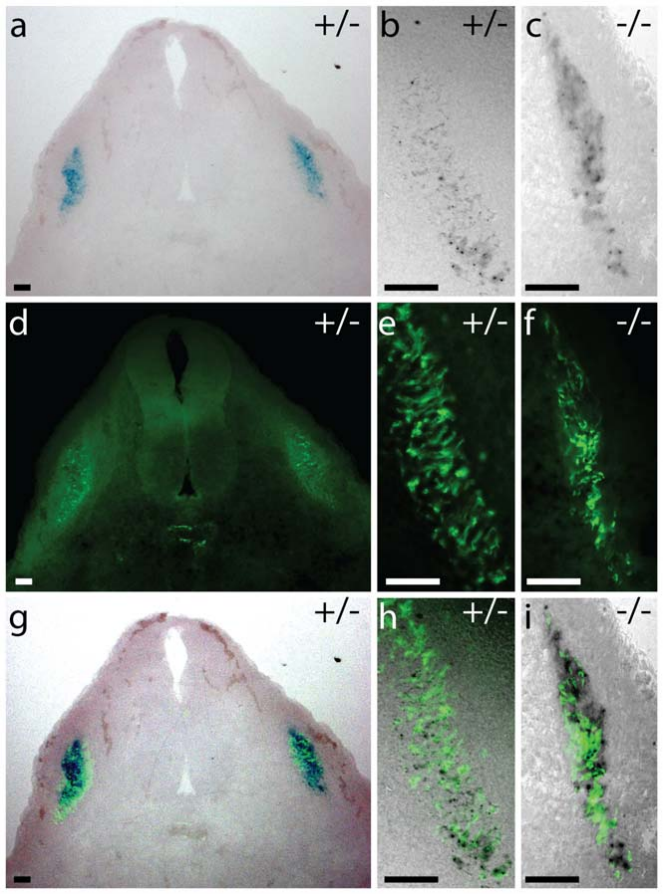
Expression of GPR126^LacZ^ in the somite. GPR126 is expressed in the somite. Heterozygous (a, b, d, e, g, h) and GPR126 null (c, f, i) embryos at 10.5 dpf were stained as whole-mount specimens for β-galactosidase activity to visualise GPR126 expression. Transverse cryostat sections of 20 µm thickness were then counter-stained with an α-SMA antibody. Note the interspersed expression of GPR126 and α-SMA with few double-positive cells. Overlay images (g–j) have been post-processed electronically to enhance visualisation. Scalebar = 50 µm.

In heterozygous GPR126^LacZ^/+ embryos, no reporter gene expression was seen up to 9.5 dpf (days post fertilisation). At 10.5 dpf a weak segmental expression is consistently observed in trunk and tail (Fig. 3 a, g) [13]. This pattern becomes more pronounced at 11.5 dpf, and rapidly fades at 12.5 dpf (Fig. 3 c, h–j). Sectioning of embryos shows the expression in close proximity to the somitic myotome, but very little lacZ expression overlaps with cells positive for a myotome marker α-smooth muscle actin (α-SMA) (Fig. 4 a, b, d, e, g, h). Notably, expression of GPR126 in each segment is restricted to very few cells in a dispersed “salt-and-pepper” fashion (Fig. 3 g, 4b). The time course of expression observed in our study is slightly delayed compared to the ISH data reported earlier [13]. This most likely reflects the time required for expression and maturation of the reporter protein.

**Figure 5:**
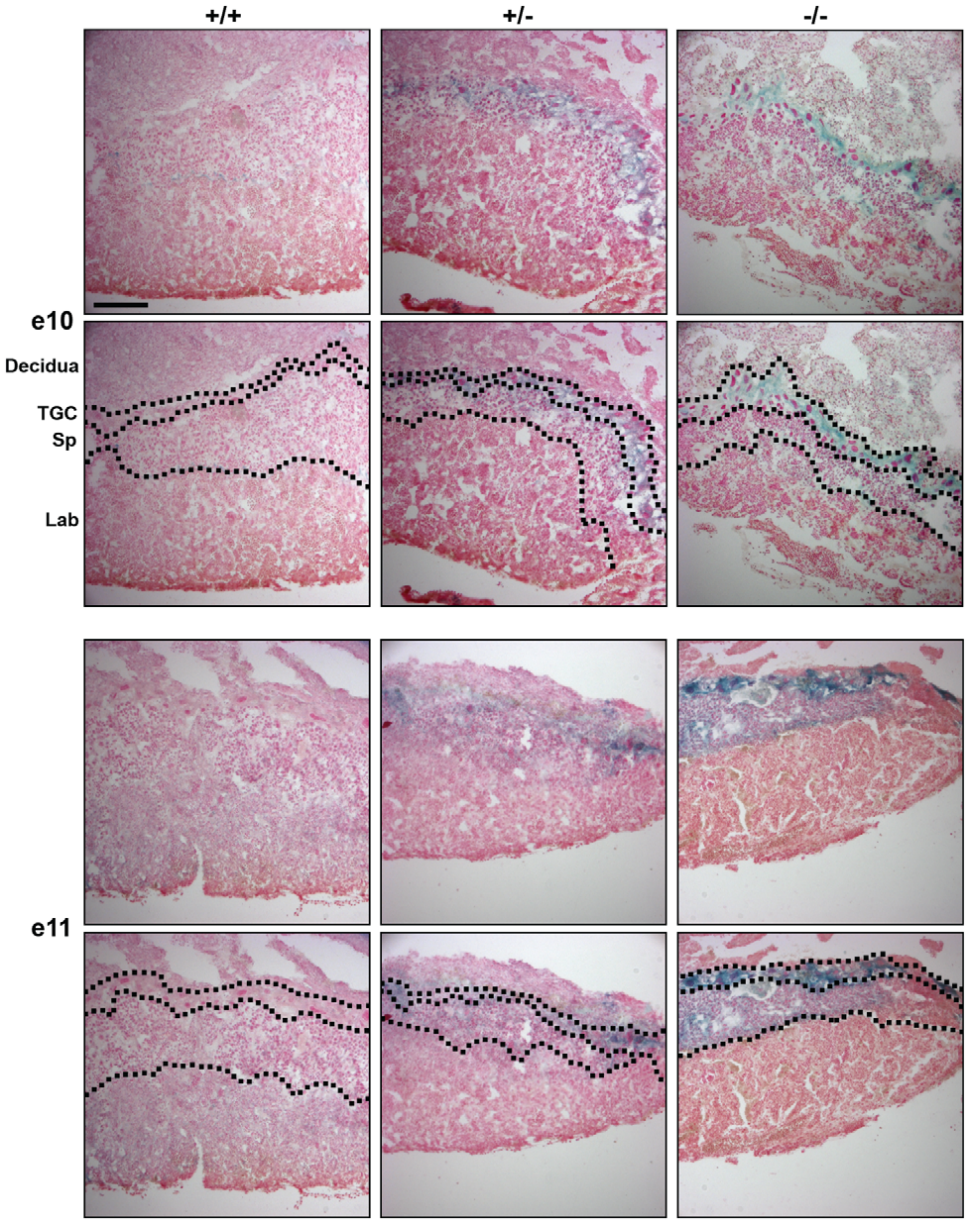
GPR126 is expressed in the placenta. *Gpr126* is expressed in trophoblast giant cells, but the anatomy of the placenta is normal in GPR126^LacZ^ homozygotes. 2 mm vibratome sections of placentas from wild-type, heterozygous and GPR126 null embryos at e10 and e11 were stained with X-Gal to visualise GPR126^LacZ^ expression, cryosectioned at 20 µm and counterstained with Nuclear Fast Red. TGC - trophoblast giant cells, Sp - spongiotrophoblast, Lab - labyrinth. Scalebar = 100 µm.

The pattern in the embryo is consistent with a very dynamic expression in a small subset of neural crest derived cells in trunk and tail. The typical locations of the cranial neural crest and the branchial arches including cardiac neural crest are negative for reporter expression. We also detected expression of GPR126^LacZ^ in the trophoblast giant cells (TGC) of the placenta (Fig. 5).

We were not able to detect clear and reproducible expression in the CNS, branchial arches and cephalic mesenchyme, the heart, vasculature, or other organ primordia of GPR126^LacZ^/+ embryos. Analysis of specimens from adult heterozygous transgenics also did not reveal distinct expression patterns of GPR126 in mature tissues. We were not able to detect a staining pattern equivalent to the expression in SCP in Zebrafish [10].

### Loss of GPR126 function leads to mid-gestation lethality

No homozygous offspring are born from matings of heterozygous carriers, indicating embryonic lethality (Table 1, 2). The genotypes of dissected embryos show that a sharp loss of viability occurs from 10.5 dpf to 12.5 dpf, consistent with the onset of intra-embryonic expression. At 11.5 dpf more than 50% (23/43) and at 12.5 dpf 80% (12/15) of the homozygous embryos are dead. No viable embryos were recovered at 13.5 dpf (Table 2).

**Table 2:** Embryonic genotypes derived from GPR126^LacZ^ matings.

Stage	Cross	+/+		+/−		−/−		resorption		N
dpf	genotypes	n(dead)	%	n(dead)	%	n(dead)	%	n	%	
**9.5**	+/− x +/−	15	23.4%	33	51.6%	14	21.9%	2	3.1%	64
**10.5**	+/− x +/−	50	24.5%	104	51.0%	35	17.2%	15	7.4%	204
**11.5**	+/− x +/−	51(3)	23.6%	95(4)	44.0%	43(23)	19.9%	27	12.5%	87
**12.5**	+/− x +/−	25(0)	22.1%	64(1)	56.6%	15(12)	13.3%	9	8.0%	87
**13.5**	+/− x +/−	19(0)	21.8%	48(2)	55.2%	2(2)	2.3%	18	20.7%	87

Genotyping of embryos dissected after timed matings reveals a sharp loss of homozygous offspring from 10.5 dpf onwards.

Mutant embryos show no obvious malformations and somite numbers are indistinguishable from controls, indicating that development is not significantly delayed at 10 dpf and 11 dpf (not shown). We did not detect abnormalities in the histological architecture of the placenta of homozygous embryos or changes in the distribution of TGC (Fig. 5). Thus, we could not find evidence for defects in placental development or function.

The number and distribution of cells expressing the GPR126^LacZ^ reporter gene is changed in homozygous embryos. An increased number of LacZ-positive cells are clustered in a narrow extension of the normal expression domain (Fig. 3 d–f, l). Histological sections confirm the presence of a larger number of LacZ-positive cells in the somitic domain, and again indicate that only a minority of these co-express α-SMA (Fig. 4 c, f, i). This observation would be consistent with a complete or partial block in the differentiation and/or migration of a GPR126-expressing cell population, leading to an accumulation of cells that might be delayed or stalled on their path of migration.

### Cardiovascular failure in GPR126 mutants

Viable homozygotes with beating hearts are regularly found in the same litter next to dead embryos showing signs of circulatory failure like congestion, edema, and internal hemorrhage. This suggests that cardiovascular function in mutants is initiated normally and fails within 24–48 hrs after the onset of GPR126 expression.

**Figure 6:**
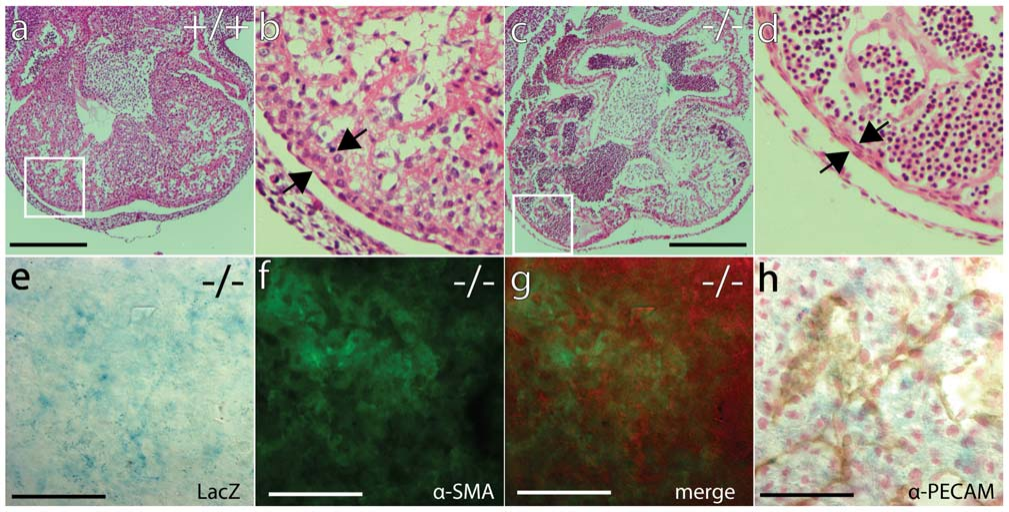
The heart phenotype of GPR126^LacZ^ mutants. (a–d) GPR126^LacZ^ mutants show dilation of the heart ventricle. Histological sections through the heart of heterozygous (a, b) and homozygous (c, d) specimens at 11.5 dpf, H&E stain. (b) and (d) are higher magnification views of the boxed area in (a) and (c). Note the thinning of the ventricular wall in (d) compared to (b) (arrows) and the presence of myocardial trabeculation. Scalebar = 100 µm. (e–h) GPR126^LacZ^ is weakly expressed in embryonic heart. Homozygous embryos at 11.5 dpf were stained for β-galactosidase activity as whole-mount specimens to visualise GPR126 expression (e), cryosectioned at 20 µm and counter-stained with an α-SMA (f) or α- PECAM (h) antibodies. In (g) the α-SMA in green is overlayed with the lacZ signal of (e) false-coloured in red, showing little overlap between the two channels. The panels (e) and (g) were post-processed electronically to enhance the visualisation of the very weak lacZ signal.

In wild-type embryos, crucial steps of cardiac development like septation and the formation of the outflow tract are only completed after 12.5 dpf. As embryos homozygous for the GPR126^LacZ^ allele die before this time point it is difficult to distinguish primary defects in heart development from secondary effects of circulatory failure due to other reasons. Expression of GPR126 could not be detected in typical cardiac neural crest (CNC) locations [20], and histological analysis indicates that septation and the formation of the CNC-derived outflow tract are initiated normally (Fig. 6).

The development of the ventricular wall is not obviously defective at 10.5 dpf, and myocardial trabeculation is formed. In homozygous mutant embryos that still showed active circulation at the time of dissection we could not detect an obvious reduction of myocardial tissue or a lack of trabeculation. However, in embryos that were dissected after onset of circulatory failure we found a pronounced thinning of the myocardial wall (Fig. 6c, d). This suggests that ventricular function is initially normal and might fail under increased load.

In heterozygous carriers of the GPR126^LacZ^ allele reporter gene expression in the ventricular wall can hardly be detected above background levels. However, the examination of myocardial tissue from homozygous mutants revealed a dispersed population of cells with a very low but reproducible level of LacZ expression (Fig. 6e). This suggests the presence of a GPR126-expressing cell population in myocardial tissue, and provides a possible rationale for cardiac failure in mutants. Counterstaining established that as in the somite only a subset of LacZ positive cells also show α-SMA expression, while no overlap could be detected with the endothelial marker PECAM (Fig. 6 f–h).

The difference in myocardial lacZ staining between heterozygotes and homozygotes could be due to the increased gene dosage in homozygotes, or due to an accumulation of lacZ positive cells in mutants. Due to the very low staining intensity the resolution of this question will require additional transgenic reagents, e.g. permanent marking of GPR126-positive cells by site-directed recombination [21], [22].

### Vascular development in GPR126 mutants

**Figure 7:**
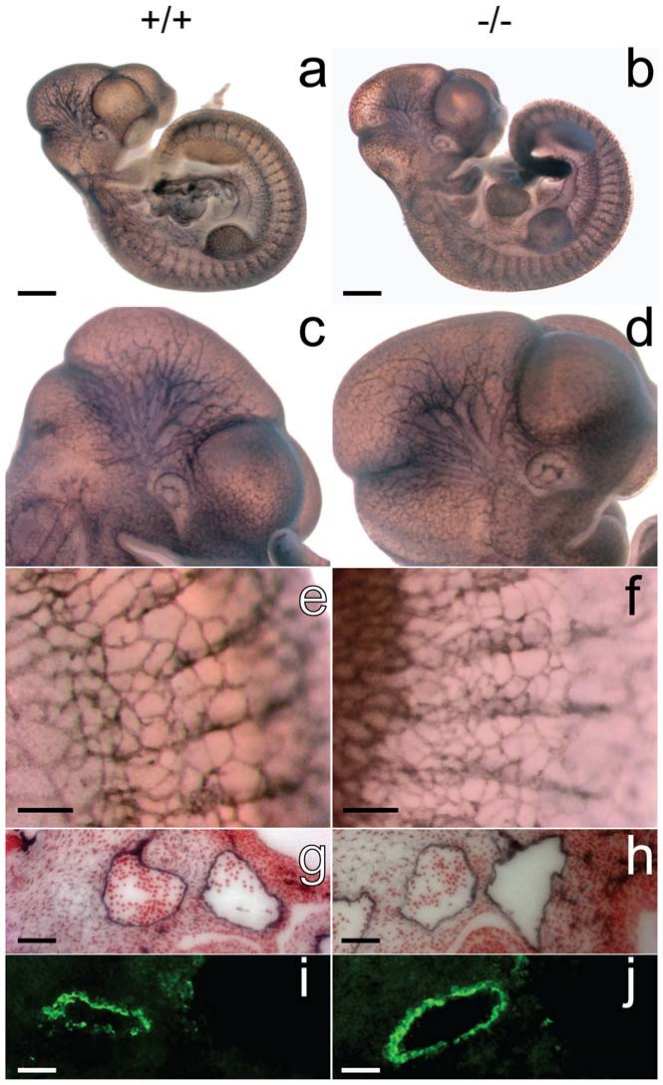
The developing vasculature in GPR126^LacZ^ embryos. Patterning of blood vessels is normal in GPR126^LacZ^ embryos. Wild-type (a, c, e, g, i) and homozygous GPR126^LacZ^ (b, d, f, h, j) embryos at 10.5 dpf were stained as whole-mount specimens with an α-PECAM (a–h) or α-SMA (i, j) antibodies. (a, b) Overall patterning of the vasculature is not disrupted in mutant embryos. (c, d) The complexity of branching of cranial vessels is normal, as is the development of intersomitic vasculature (e, f). (g, h) The dorsal aortae form normally and smooth muscle cells are recruited to stabilize the vessels (i, j). Scalebar1 mm (a, b) 200 µm (e, f) and 50 µm (g–j).

GPR126 was initially isolated from HUVEC cultures that had been challenged with lipopolysaccharides or thrombin [14] and was implicated in endothelial cell function. We observed intra-embryonic hemorrhage in ∼50% (24/49) of GPR126^LacZ^ embryos that were found dead at dissection, and therefore investigated defects in vascular development as a possible cause of lethality. Immuno-histochemical detection of the endothelial marker PECAM-1 in whole-mount embryos and in histological sections did not reveal any consistent defects of angiogenesis or vasculogenesis in mutant embryos (Fig. 7). We could not detect co-expression of GPR126^LacZ^ and PECAM, indicating that the expression level is below the detection limit in unchallenged endothelial cells.

The development of endothelium in large and small vessels (Fig. 7 a, b, e, f) and the complexity of the vascular tree (Fig. 7 c, d) are not significantly different between homozygous mutants and controls. No abnormalities were detected in placental vessels and umbilical cord. The paired dorsal aortae develop and fuse normally (Fig. 7 g, h), and the vascular wall is stabilised by the recruitment of smooth muscle cells (Fig. 7 i, j) and extracellular matrix. Hematopoietic clusters in mutant vessels also develop at the correct time and localisation (not shown). Thus, in spite of the reported expression of GPR126 in endothelial cell cultures we could not detect expression of GPR126 in endothelia *in vivo*, and were not able to find evidence supporting a defect of vascular development as the cause of embryonic lethality.

## Discussion

We describe the first genetic analysis of GPR126 function in a mammalian model. The targeted mutation GPR126^LacZ^ deletes part of the 7TM region (Fig. 1a) and is presumably a null allele. Residual wild-type transcripts could not be detected in mutant embryos, and a truncation of the related receptor *lat-1* has been shown to lack residual function in C. elegans [9], [23].

Homozygous mutant embryos show fully penetrant lethality due to cardiovascular failure at mid-gestation. Lethality coincides with the expression of GPR126^LacZ^ in a small group of cells in trunk and tail somites, but due to the very low and transient levels of gene expression we could not directly determine which anatomical structures are ultimately derived from this cell population. We did not find evidence for developmental defects of the vascular system, the cardiac neural crest, or the placenta, and suggest that GPR126 is required for the development and/or the function of nascent myocardial tissue.

Expression of human GPR126 has been reported *in vitro* in HUVEC cultures stimulated by lipopolysaccharide (LPS) [14]. However, we could not detect GPR126^LacZ^ in embryonic endothelia *in vivo* and did not observe morphological abnormalities of the developing vascular system. A defect of the cardiac neural crest in GPR126 mutants also appears unlikely. Firstly, CNC cells migrate via the branchial arches [24], where GPR126^LacZ^ is not expressed. Secondly, the development of the cardiac outflow tract is initiated normally in GPR126 homozygotes. Thirdly, mice with severe disruption of CNC development, e.g. *Splotch* mutants, show malformation of the outflow tract by 13.5 dpf and embryonic death by 14.5 dpf [24], significantly later than GPR126 mutants. This strongly suggests that abnormal development of the CNC would not be sufficient to explain the phenotype of GPR126^LacZ^ homozygotes.

Although GPR126 is expressed in TGC, the histological architecture of the placenta is normal in GPR126 homozygotes. Embryonic development up to 11 dpf is not delayed, arguing against a significant impairment of placental function. We could not find precedents in the literature where a functional defect in a morphologically normal placenta causes fully penetrant lethality in a narrow time window at 11.5–12.5 dpf, as observed in GPR126 mutants. The lethality profile is the same on inbred and outbred genetic backgrounds, indicating that a loss of immune privilege is not a likely cause of embryonic death. In future experiments, the role of GPR126 in the placenta could be directly tested by creating chimeras of homozygous GPR126 embryos with tetraploid wild-type embryos.

Weakly LacZ-positive cells were observed in the ventricular wall of GPR126^LacZ^ homozygotes, suggesting that GPR126 might have a function in myocardial development. In GPR126 mutants the myocardial wall is thinned, but shows no defect in trabeculation. Thus, a defect in the development of the ventricle wall of the heart appears to be the most likely cause of death in GPR126 mutants.

In contrast to the embryonic lethality in GPR126^LacZ^ mice, zebrafish homozygous for the *gpr126(st49)* allele develop into fertile adults with a defect in Schwann cell function [10]. The predicted products of GPR126^LacZ^ and *gpr126(st49)* are similar (Fig. 1a) [10], arguing against an allele-specific effect. Due to the early embryonic lethality we could not investigate the role of GPR126 in mouse SCP and myelin sheath formation. However, it is unlikely that a defect in SCP causes lethality in GPR126^LacZ^ mice, as other mutants lacking SCP develop to term [25].

Our results indicate that GPR126 has an essential function in mammalian development that has not been predicted by the analysis of GPR126 mutations in Zebrafish. This could either be due to true differences in the developmental pathways of fish and mammals, or due to different levels of functional redundancy and compensation by other members of the Adhesion-GPCR family.

A link between heart morphogenesis and Schwann cell development is demonstrated in mice mutant for components of the neuregulin/erbB2-4 pathway, which display a syndrome of myocardial failure and defects in SCPs [26], [27], [28]. In zebrafish, the mutant phenotypes are milder with respect to embryonic lethality, presumably due to the existence of additional paralogs, e.g. two copies of erbB3 (erbB3a and erbB3b [29], [30]). This suggests that the underlying developmental pathways are similar between fish and mammals, and that partial genomic redundancy is the cause of phenotypic differences.

The conserved synteny at the GPR126 locus clearly indicates that mouse and zebrafish GPR126 are true orthologs. We could not detect an additional copy of GPR126 in the zebrafish genome which could explain the milder phenotype of the *gpr126(st49)* allele. However, the most closely related Adhesion-GPCR, GPR112, shows much higher homology to GPR126 in fish than in mammals (Fig. 1a). This suggests that in zebrafish a partial redundancy between GPR126 and GPR112 might ameliorate the phenotype of GPR126 mutants. Experimentally this could be tested by knockdown of GPR112 in *gpr126(st49)* mutants.

Conditional inactivation of the erbB2 pathway in adult mice leads to dilated cardiomyopathy and has been linked to the cardiotoxicity of the therapeutic antibody trastuzumab (Herceptin®) [31]. Our results strongly suggest that any therapeutic intervention targeting GPR126, e.g. to modulate myelination, will require careful consideration of cardiac side effects.

Due to the very restricted and transient expression of GPR126, a limitation of our study has been the difficulty to characterise the origin and fate of GPR126-positive cells. We consider it unlikely that standard ISH and IF will be able to fully resolve this problem. The further analysis of GPR126 function will require the identification of the tissues formed by GPR126-positive cells by permanent transgenic marking, e.g. by constitutive activation of a LacZ reporter gene by a GPR126-Cre transgene [21], [22], the use of tissue specific conditional mutagenesis, and the analysis of chimeric embryos.

Genetic variation at the GPR126 locus is linked to the heritability of height in the human population [15], [16], [17]. The molecular nature of different GPR126 alleles is not known. GPR126 contributes specifically to trunk length rather than limb length, consistent with our finding that the gene is expressed in a segmental pattern in a putative migratory cell population. While our findings cannot explain the role of GPR126 polymorphisms in humans, they might guide further mechanistic studies.

## Materials and Methods

### Ethics statement

All procedures were carried out in accordance with the Animals (Scientific Procedures) Act 1986 and with the approval of local ethical review committees under the UK Home Office Project License PPL 30/2462.

### Comparative genomic analysis

Genomic loci and predicted gene products for GPR126 were obtained from the Ensembl database (http://www.ensembl.org) and analysed with the Geneious software package (Biomatters Ltd., Auckland, NZ). The zebrafish GPR112 locus was identified by GNOMON annotation of the genomic scaffold 1269 (Zv8, NCBI Acc NW_001877055) on Chr. 10. The predicted mRNA and protein are available under NCBI Acc XM_002663550 and XP_002663596, and are supported by EST evidence.

### Analysis of RNA expression

Embryos were dissected from the uterus, the yolk sac and amnion removed, and the embryo and placenta separated. Embryos were minced on ice in sterile phosphate buffered saline (PBS) and stored in RNALater at −20°C. Tissues and embryos were homogenised in a ground glass homogeniser and total RNA was isolated with Trizol reagent (Invitrogen) according to manufacturer's protocol. First strand cDNA was synthesized with Superscript III reverse transcriptase (Invitrogen) using random hexamer primers (Promega).

RT-PCR was performed according to standard protocols. Relative cDNA levels were determined by PCR for Hypoxanthine-guanine phosphoribosyltransferase (*Hprt*) and β-actin. The primer sequences are: mGPR126: 5′-CTTGTCCTGTCCAATCCTTCCGGT-3′, 5′-TCCACCGCTATAT TCTAAAATTCTG-3′, 5′-CAGCAACTCTGATGTAGCTGGCGT-3′, and 5′-GGTCAGTTTTGA CAGGGACTTG-3′; HPRT: 5′-ACTTGCTCGAGATGTCATGAAGG-3′ and 5′-CCTGTATCCAA CACTTCGAGAGGT-3′; β-actin: 5′-GCCCAGAGCAAGAGAGG TATCCT-3′, and 5′-CGTCTC CGGAGTCCATCACAA-3′.

### Generation of the GPR126^LacZ^ allele

For timed matings, females were housed with stud males and checked for vaginal plugs each morning, with the morning of plug discovery taken as embryonic day 0.5 (0.5 dpf).

The vector for homologous recombination was constructed as described previously [19], [32]. Homology arms of 3924 bp (5′) and 1256 bp (3′) were generated by PCR and inserted into a targeting vector providing the reporter gene and the positive and negative selection cassettes. In the targeted locus, most of exon 18 is deleted and replaced by a 4.5 kb fragment containing the IRES-LacZ-neo^R^ cassette, resulting in a truncation of GPR126 at residue 830 immediately N-terminal of the first transmembrane helix.

The vector was electroporated into the CCB embryonic stem cell line, and correctly targeted clones identified by southern blotting and PCR. The targeted allele GPR126^LacZ^ was transmitted through the germ line and maintained on inbred 129SvEv and outbred MF1 backgrounds, as well as back-crossed to C57B/6 for 10 generations. Mice and embryos were genotyped by PCR with internal control. A common reverse primer binds wild-type and targeted alleles, while forward primers bind the neomycin resistance cassette or wild-type mGPR126 sequence located 5′ to the β-galactosidase/neomycin cassette yielding products of 180 bp or 266 bp respectively. DNA was extracted from ear-biopsies or yolk-sacs, and PCR was performed with the primers 5′-ATGAGAAACATGAGGATTAAAGGGGA-3′, 5′-GGATCTTCCAAGGAGTGCCTCAC AA-3′ for mGPR126, and 5′- GCAGCGCATCGCCTTCTATC-3′ for the neomycin resistance cassette.

### Whole-mount embryo staining

Histochemical staining for LacZ reporter gene activity was performed as described [19]. Adult tissues or embryos dissected after timed matings were washed twice for 5 min in PBS containing 2 mM magnesium chloride, fixed at 4°C in 0.2% (v/v) glutaraldehyde, 100 mM phosphate buffer pH 7.4, 5 mM EGTA, 2 mM magnesium chloride, and washed three times for 5 min at room temperature in LacZ Wash Buffer (100 mM phosphate buffer pH 7.4, 0.01% (v/v) Nonidet P-40, 0.02% (w/v) sodium deoxycholate, 2 mM magnesium chloride). Embryos and tissues were incubated for 15–48 h in LacZ stain (0.8–1.6 mg/ml X-Gal, 100 mM phosphate buffer pH 7.4, 0.01% (v/v) Nonidet P-40, 0.02% (w/v) sodium deoxycholate, 5 mM potassium ferricyanide, 5 mM potassium ferrocyanide, 2 mM magnesium chloride) at 37°C in the dark. Samples were post-fixed in 4% (w/v) paraformaldehyde in PBS and Histology.

For whole-mount immunohistochemistry, embryos were stained with primary antibodies against PECAM and αSMA (rat anti-mouse PECAM clone MEC13 (BD Pharmingen) at a 1 in 200 dilution and mouse anti-human αSMA clone 1A4 (Dako) at a 1 in 500 dilution) and HRP conjugated secondary antibodies against rat and mouse (goat anti-rat IgG (Stratech) at a 1 in 100 dilution and goat anti-mouse IgG (Millipore) at a 1 in 100 dilution). Embryos were fixed in 4% (w/v) paraformaldehyde in PBS, dehydrated through a series (25%, 50%, 75%, 100%, 100% (v/v)) of methanol in PBS, and stored at −20°C.

Specimens were bleached in 6% (v/v) hydrogen peroxide in methanol for 5 h 30 min on a shaking platform, rehydrated through a series (100%, 75%, 50%, 25% (v/v)) of methanol in PBS, permeabilised in PBS containing 0.1% (v/v) Triton X-100, and blocked in blocking solution (PBS containing 2% (w/v) Marvel skimmed milk powder and 0.1% (v/v) Triton X-100) for 1 h before incubation in rat anti-mouse PECAM clone MEC13 primary antibody (BD Pharmingen) at a 1 in 200 dilution in blocking solution. Specimens were then incubated twice in blocking solution for 1 h at 4°C and three times for 1 h at room temperature before incubation in HRP conjugated goat anti-rat IgG secondary antibody (Stratech) at a 1 in 100 dilution in blocking solution overnight at 4°C. After further incubations in blocking solution for 1 h at 4°C and three times for 1 h at room temperature before further blocking in PBS containing 0.2% (w/v) bovine serum albumin and 0.1% (v/v) Triton X-100, once for 10 min at room temperature, once for 40 min at room temperature, the stain was developed with PBS containing 0.5 mg/ml DAB, 2 mg/ml ammonium nickel sulphate and 0.003% (v/v) hydrogen peroxide for 2–10 min.

### Histology

For frozen sections, samples were cryoprotected in 30% (w/v) sucrose in PBS and embedded in TissueTek (Bayer). 10–20 µm sections were taken using a Bright 5040 cryostat. For paraffin-embedded sections, 6 µm sections were taken on a Leica DSC1 microtome and mounted onto X-tra adhesive slides (Surgipath). Haematoxylin/eosin staining was performed according to standard protocols.

Immunohistochemistry was performed according to standard protocols with the primary antibodies α-GPR126 (Abcam) 1∶50, 1∶100 and 1∶500 dilution, α-PECAM (platelet and endothelial cell adhesion molecule) clone MEC13 (BD Pharmingen) 1∶200 dilution, α-smooth muscle actin (αSMA) clone 1A4 (Dako) 1∶500 dilution. Signal was detected with secondary antibodies conjugated to horseradish peroxidase (goat anti-rabbit IgG (Millipore), goat anti-rat IgG (Stratech), horse anti-mouse IgG (Vector Laboratories)), or a fluorophore (Alexa Fluor 488 conjugated goat anti-mouse IgG (Invitrogen), all in 1∶100 dilution. Sections were counterstained with Nuclear Fast Red or Haematoxylin and mounted using Vectamount or Vectashield (Vector Laboratories).

### Microscopy

Histological sections were visualised with a Zeiss Axioplan2.0 microscope fitted with an AxioCam HD camera. Whole-mount samples were documented with a Nikon SMZ1500 dissection microscope fitted with a Leica DC500 camera. Images were collected and manipulated using Adobe Photoshop 9.0.

## Supporting Information

Figure S1 Alignment of zebrafish GPR126 and GPR112, showing the high degree of homology between the paralogs.
(0.66 MB TIF)

Figure S2 Expression of Gpr126 in adult tissues. cDNA was amplified from wild-type mouse organs using gene specific primers against Gpr126. β-Actin was amplified as positive control to normalize cDNA concentration (β-Actin + RT (reverse transcriptase)). β-Actin -RT: reaction without reverse transcriptase as control for genomic DNA contamination. Expected band sizes: Gpr126 759bp, β-Actin 303bp.
(0.79 MB TIF)
