# Supplementary figures and images for: The Orphan Adhesion-GPCR GPR126 Is Required for Embryonic Development in the Mouse
Source: PLoS One. 2010 Nov 18;5(11):e14047. doi: 10.1371/journal.pone.0014047 (PMC2987804; doi:10.1371/journal.pone.0014047)

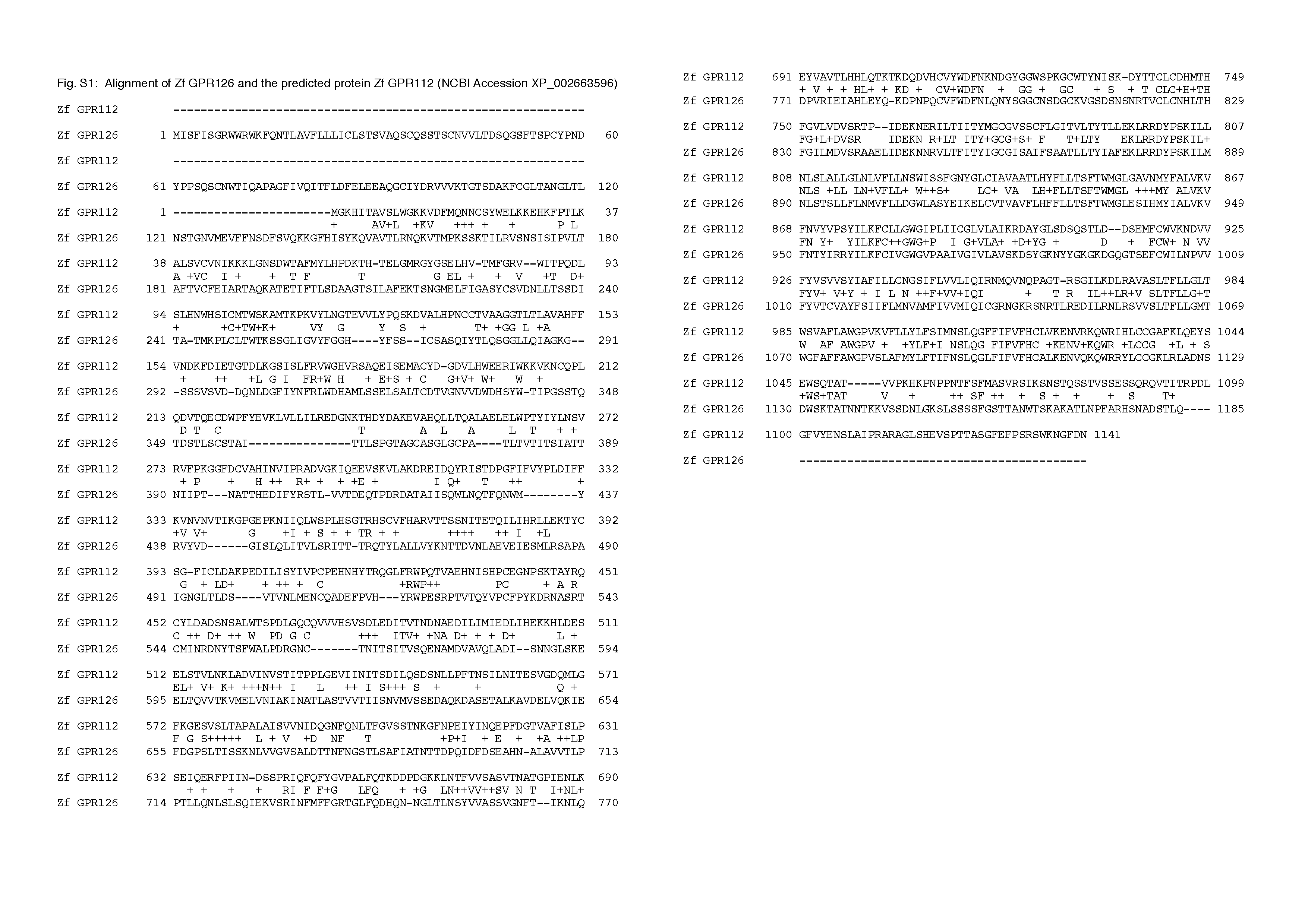

Supplement: Figure S1 — Alignment of zebrafish GPR126 and GPR112, showing the high degree of homology between the paralogs. (0.66 MB TIF) [file pone.0014047.s001.tif]

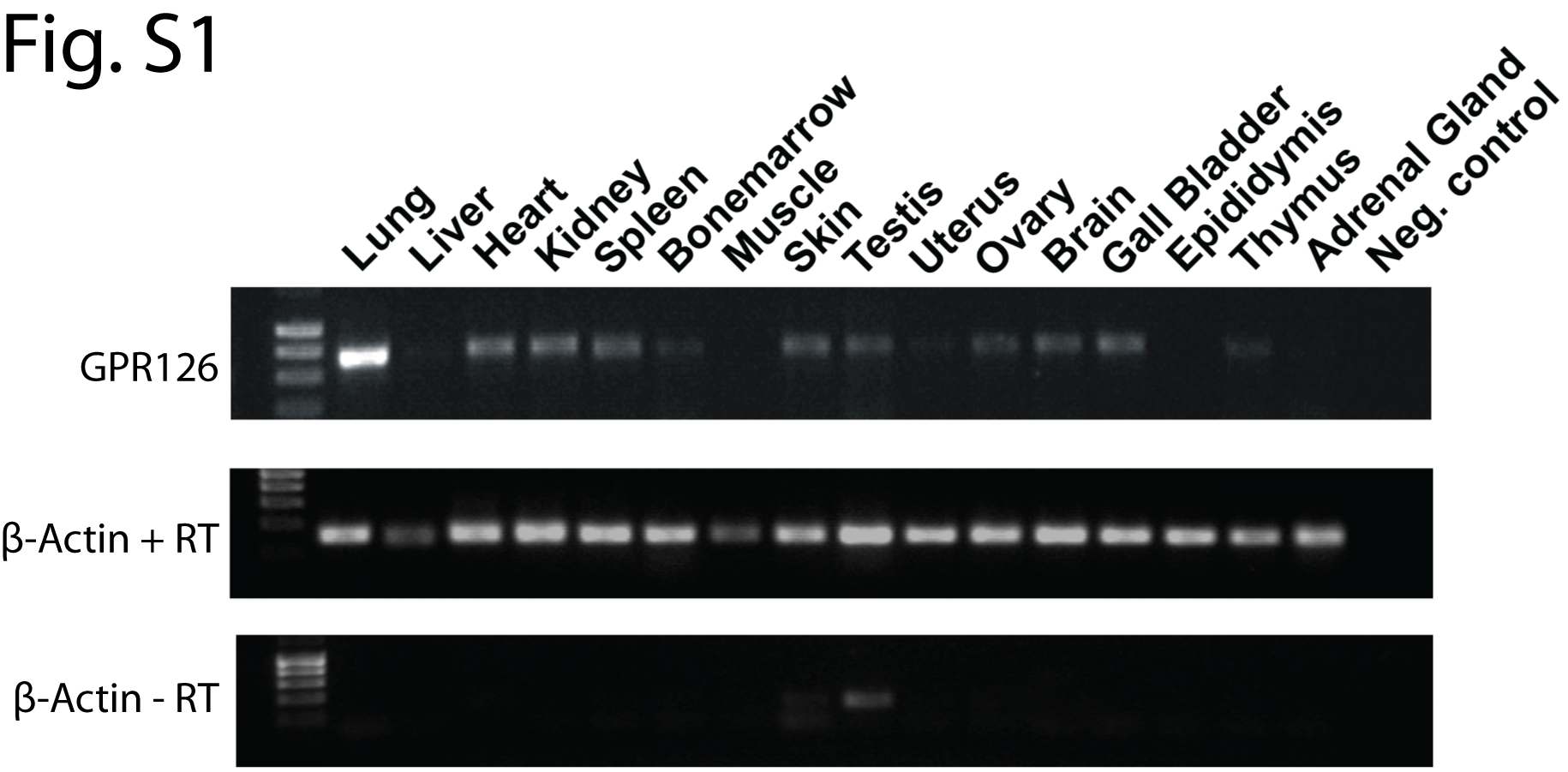

Supplement: Figure S2 — Expression of Gpr126 in adult tissues. cDNA was amplified from wild-type mouse organs using gene specific primers against Gpr126. β-Actin was amplified as positive control to normalize cDNA concentration (β-Actin + RT (reverse transcriptase)). β-Actin -RT: reaction without reverse transcriptase as control for genomic DNA contamination. Expected band sizes: Gpr126 759bp, β-Actin 303bp. (0.79 MB TIF) [file pone.0014047.s002.tif]
